# The clock is ticking: the impact of ageing on T cell metabolism

**DOI:** 10.1002/cti2.1091

**Published:** 2019-11-18

**Authors:** Kylie M Quinn, Riya Palchaudhuri, Clovis S Palmer, Nicole L La Gruta

**Affiliations:** ^1^ School of Health and Biomedical Sciences RMIT University Bundoora VIC Australia; ^2^ Department of Biochemistry Biomedicine Discovery Institute Monash University Clayton VIC Australia; ^3^ Life Sciences Macfarlane Burnet Institute for Medical Research and Public Health Melbourne VIC Australia; ^4^ Department of Infectious Diseases Monash University Melbourne VIC Australia; ^5^ Department of Immunology and Pathology Monash University Melbourne VIC Australia

**Keywords:** ageing, cell signalling, immunosenescence, metabolism, T cell

## Abstract

It is now clear that access to specific metabolic programmes controls the survival and function of various immune cell populations, including T cells. Efficient naïve and memory T cell homoeostasis requires the use of specific metabolic pathways and differentiation requires rapid and dramatic metabolic remodelling. While we are beginning to appreciate the crucial role of metabolic programming during normal T cell physiology, many of the potential impacts of ageing on metabolic homoeostasis and remodelling in T cells remain unexplored. This review will outline our current understanding of T cell metabolism and explore age‐related metabolic changes that are postulated or have been demonstrated to impact T cell function.

## Graphical abstract

Specific metabolic programmes control the survival and function of T cells. As we age, T cell responses can become highly dysfunctional, but the impact of ageing on the T cell metabolism remains to be fully understood. This review summarises our current understanding and explores age‐related metabolic changes that are postulated or have been demonstrated to impact T cell function.

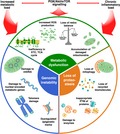

## Introduction

As we age, our ability to mount robust T cell responses declines because of shifts in both naïve and memory T cell compartments. This has been more extensively reviewed by Goronzy & Weyand^1^ and Nikolich‐Zugich,^2^ but briefly, the thymus involutes with age and naïve T cells thereafter rely on homoeostatic proliferation to maintain numbers. Access to IL‐7‐rich niches in the lymph node decreases^3^ and the frequency and number of naïve T cells therefore declines. This decline is more marked for CD8 T cells as compared to CD4 T cells,^4^ leading to a predominance of CD4 T cells with increasing age. As a result of the decline in overall number, T cell receptor (TCR) clonal diversity is reduced and there is evidence that T cells with more self‐reactive TCRs are selectively retained during ageing.^5^, ^6^, ^7^ In contrast to the loss of naïve T cells, ageing leads to an accumulation of memory T cell subsets, many of which are dysfunctional, such as exhausted, terminally differentiated or T effector memory cells that re‐express CD45RA (T_EMRA_) cells. TCR clonal diversity of memory cells may become skewed in older individuals with massive expansions of cells specific for chronic infections, such as cytomegalovirus (CMV).^8^ Critically, the ability of both naïve and memory T cell populations to proliferate in response to TCR stimulation declines with age, with memory‐phenotype cells being more susceptible to this loss of function.^5^, ^9^ The cumulative impact is that T cell responses are substantially delayed and reduced in older individuals,^10^ which leads to diminished vaccine efficacy and can leave us vulnerable to infections and cancer.

To improve T cell responses in older individuals, we must define the molecular mechanisms that limit these responses and immuno‐metabolism is emerging as an important but under‐examined mechanism. Immuno‐metabolism uses core principles of immunology and metabolism to identify metabolic pathways that regulate specific functional outcomes of immune cells. Much of this work has been performed with immune cells from young individuals or mice, but more recent research now aims to extend these principles and observations to the ageing immune system.

Lifestyle modifications already suggest that metabolic alterations contribute to age‐related T cell dysfunction. Exercise and caloric restriction both induce marked effects on cellular metabolic activity, likely by limiting oxidative stress, altering lipid metabolism and inducing mitochondrial biogenesis.^11^ These interventions can slow biological ageing in general, but exercise and caloric restriction also specifically improve T cell longevity and function with age.^12^, ^13^ In addition, exercise was recently linked with increased thymic output in older individuals.^14^ Collectively, this strongly implicates metabolic alterations in the development of age‐related T cell dysfunction.

## The fundamentals of T cell metabolism

**Figure 1:**
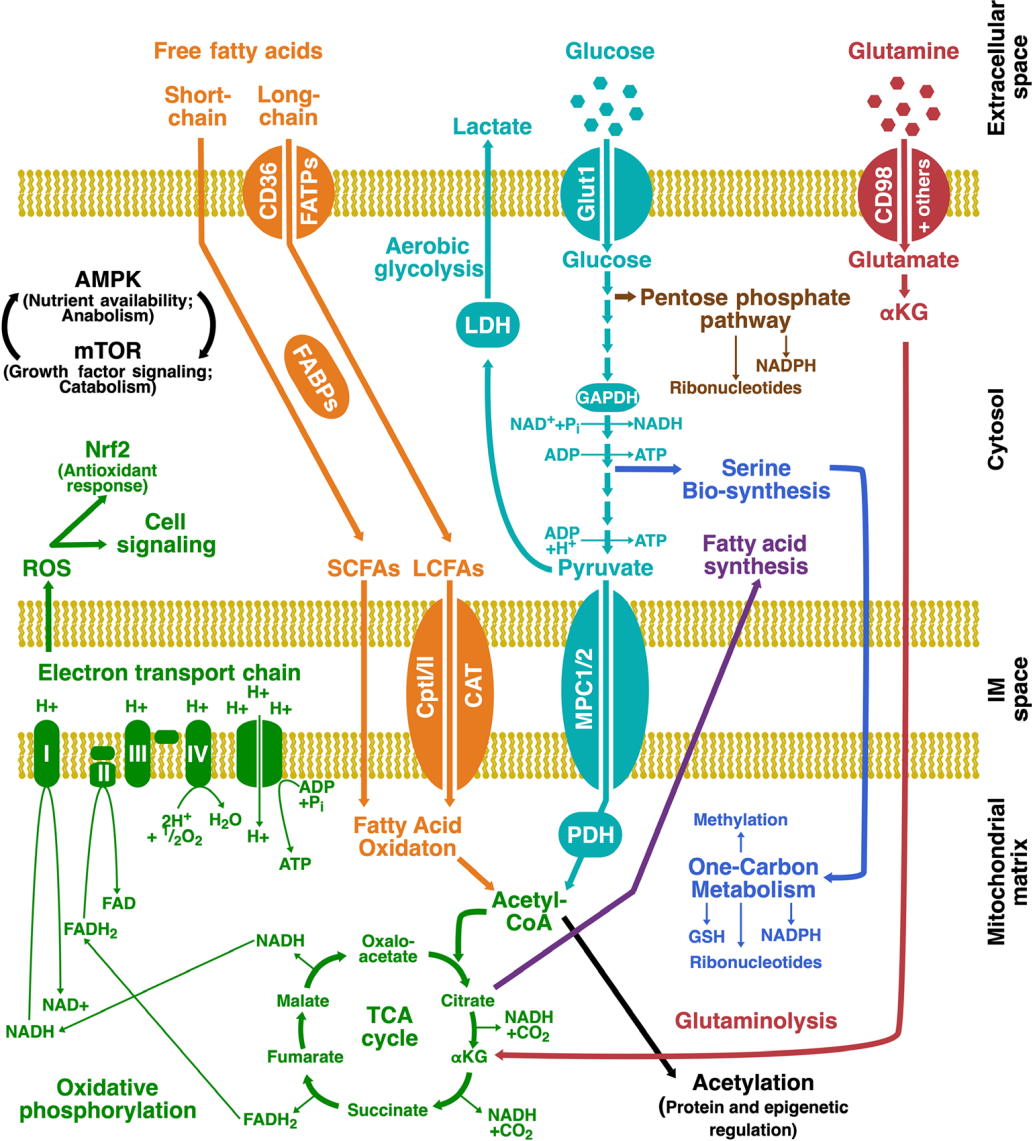
Schematic of basic metabolic pathways used in T cells. (Green) In oxidative phosphorylation (OXPHOS), the tricarboxylic acid (TCA) cycle reduces redox cofactors, nicotinamide adenine dinucleotide (NAD^+^) and flavin adenine dinucleotide (FAD), to generate NADH, FADH_2_ and CO_2_. NADH and FADH_2_ donate electrons to drive the electron transport chain (ETC). The ETC shuttles electrons through complexes I–IV, driving progressive export of protons into the intermembrane space of the mitochondria to establish a proton gradient, and terminating in the consumption of O_2_ and generation of H_2_O. The proton gradient (mitochondrial membrane potential) drives complex V [adenosine triphosphate (ATP) synthase] to generate ATP. The ETC can generate ROS, which can promote TCR signalling through NFAT and can trigger an antioxidant response through nuclear factor erythroid 2‐related factor 2 (Nrf2). (Orange) Upstream of FAO, short‐chain fatty acids diffuse across cellular membranes, but long‐chain fatty acids are actively taken up by the cell through transporters such as CD36 and fatty acid transport proteins (FATPs) and shuttled in the cytosol by fatty acid binding proteins (FABP). Long‐chain fatty acids are modified and imported into the mitochondrial matrix by Cpt1, CptII and CAT, where FAO takes place to generate acetyl‐CoA that can enter the TCA cycle. (Purple) In FAS, citrate is withdrawn from the TCA cycle to generate fatty acids and lipids for storage in the cytosol. (Red) In glutaminolysis, glutamine is taken up by the cell via the glutamine transporters, such as CD98, converted to glutamate and then α‐ketoglutarate, which can enter the TCA cycle. (Aqua) In glycolysis, extracellular glucose is taken up via glucose transporters, such as Glut1, and subsequently processed in the cytosol to yield ATP, NADH and pyruvate. Pyruvate can either be (1) transported into the mitochondrial matrix by the mitochondrial pyruvate transporters (MPC) 1 and 2, where the PDH complex converts it to acetyl‐CoA to fuel the TCA cycle and OXPHOS, or (2) diverted away from the mitochondria, converted to lactate by LDH and exported from the cell as lactic acid, in a pathway variant known as ‘aerobic glycolysis’. (Brown) The PPP diverts metabolic intermediates of glycolysis for the synthesis of NADPH and ribonucleotides. (Blue) One‐carbon metabolism uses glycine or serine, which can also be diverted from metabolic intermediates of glycolysis, for biosynthesis of nucleotides, lipids, NADPH, GSH and substrate for methylation reactions. (Black) Engagement of these metabolic pathways is controlled by reciprocal regulation of AMPK and mTOR. In addition, metabolic intermediates such as acetyl‐CoA can be used to control activation of proteins and acetylation of histones.

There are many complex metabolic processes that regulate cellular biology, but here we briefly introduce key pathways for T cell activation, function and survival; oxidative phosphorylation (OXPHOS), amino acid metabolism, fatty acid oxidation (FAO), fatty acid synthesis (FAS), glycolysis, the pentose phosphate pathway and one‐carbon metabolism (Figure 1; reviewed in Almeida *et al*.,^15^ Wang and Green,^16^ Ron‐Harel *et al*.^17^ and Palmer *et al*.^18^). These pathways are crucial for generating adenosine triphosphate (ATP) for energy, and for providing metabolites for auxiliary processes such as macromolecule (DNA, protein, lipids) synthesis, protein post‐translational modifications, epigenetic modifications and in cell signalling. As a general rule, quiescent T cells use catabolic pathways, which use substrates very efficiently for energy output, while activated T cells engage anabolic pathways, which can be energetically inefficient but allow the cell to build biomass to support protein production and cell division.

The pivotal pathway by which energy is generated in T cells is located in the mitochondria,^19^ where the tricarboxylic acid (TCA) cycle is coupled to the electron transport chain (ETC), which permits OXPHOS to generate ATP (Figure 1). The TCA cycle can be fuelled by a number of different substrates, including pyruvate, fatty acids and amino acids. Importantly, OXPHOS depends on ready access to the reduction‐oxidation (redox) cofactors, nicotinamide adenine dinucleotide (NAD^+^) and flavin adenine dinucleotide (FAD) and the integrity of the mitochondria to build mitochondrial membrane potential for the action of ATP synthase. Ultimately, OXPHOS is a highly efficient way by which cells generate energy.

Key substrates for the TCA cycle can be derived from fatty acids and amino acids. Specifically, resting T cells can use FAO (beta oxidation) to fuel the TCA cycle^20^ (Figure 1). Fatty acids are transported into the mitochondrial matrix by a complex containing carnitine palmitoyltransferase Ia, where they undergo FAO to generate acetyl‐CoA that can enter the TCA cycle. Notably, FAO is a very efficient way to generate energy, which is advantageous during quiescence when energy demands are low. Activated T cells engage in glutaminolysis^21^ to enable anaplerosis, whereby metabolic intermediates are supplemented into the TCA cycle to compensate intermediates that are removed for other biosynthetic pathways, such as FAS described below (Figure 1). Glutamine can be taken up by the cell through a number of transporters, including CD98 (Slc7a5), SNAT1/2 (Slc38a1/2) and others, and converted to glutamate and then α‐ketoglutarate, which can enter the TCA cycle. This is advantageous as glutamine is an abundant amino acid in plasma and glutaminolysis can also promote homoeostasis of redox cofactors and donates carbon and nitrogen to macromolecules. Substrates can also be withdrawn from the TCA cycle in certain circumstances. Acetyl‐CoA can be diverted for acetylation of proteins or histones to regulate protein activity and DNA accessibility, respectively. Citrate can be withdrawn in activated and memory T cells for FAS, to generate lipids in the cytosol^20^ (Figure 1). FAS allows the cell to generate new cellular membrane or build lipid droplet stores for subsequent energy needs and may promote proliferation and survival.

Glycolysis is another key cellular pathway for energy generation (Figure 1). Extracellular glucose is taken up via glucose transporters, such as Glut1, and subsequently processed in the cytosol to yield a small amount of ATP, NADH and pyruvate. Pyruvate can be transported into the mitochondria and converted into acetyl‐CoA by the pyruvate dehydrogenase (PDH) complex to fuel the TCA cycle and OXPHOS. However, cells that are rapidly proliferating, such as activated T cells, can also engage in aerobic glycolysis,^22^ where some pyruvate is diverted away from the mitochondria, converted to lactate and exported from the cell as lactic acid. This diversion is bioenergetically inefficient in terms of ATP production (aerobic glycolysis generates two molecules of ATP per molecule of glucose, while glycolysis coupled to OXPHOS generates 34 molecules of ATP per molecule of glucose), but it enables more rapid transit of glycolytic intermediates through the glycolytic pathway. These glycolytic intermediates can be used in other critical auxiliary pathways.

One such auxiliary pathway is the pentose phosphate pathway (PPP; Figure 1). The PPP drives synthesis of NADPH, an important reducing agent for glutathione (GSH), and ribonucleotides, to generate new DNA or RNA during periods of rapid cell division.^16^ Another important pathway is one‐carbon metabolism, which is a broad set of reactions that occur both in the cytosol and mitochondria and contribute to biosynthesis of nucleotides, lipids, NADPH, GSH and substrate for methylation reactions^23^ (Figure 1). Substrate for one‐carbon metabolism is derived either from serine or glycine. Serine starvation can limit T cell proliferation^24^, ^25^ and serine biosynthesis from glycolytic intermediates may be a key substrate for one‐carbon metabolism during T cell activation. Given the role of these auxiliary pathways in biosynthesis of cellular macromolecules and support of redox balance, they are regarded as pivotal for cellular homoeostasis during times of rapid cell division.

## Regulation of metabolism

To meet a sudden increase in energy demand, a cell can either increase flux through existing metabolic machinery (which is known as the spare respiratory capacity) or generate new metabolic machinery.

Mitochondria are essential metabolic machinery and new mitochondria are generated in a process called mitochondrial biogenesis. Activation of the transcription factors, nuclear factor erythroid 2‐related factor 1 (Nrf1) and PPARγ coactivator‐1α can drive expression of nuclear‐encoded mitochondrial genes to facilitate mitochondrial biogenesis. It should be noted that mitochondria are very dynamic and can fragment into discrete organelles or fuse into larger structures.^26^ Fusion can facilitate more efficient energy production but it can also be a stress response. If mitochondria have damaged copies of mitochondrial DNA (mtDNA), they can fuse into larger structures to access undamaged copies of mtDNA, in a process known as complementation.^27^ Fragmentation or fusion is mediated by dynamin‐related protein 1 or mitofusin 1 and 2 and Optic atrophy 1 (Opa1), respectively,^26^ and these processes can shape T cell differentiation. Memory T cells have more fused mitochondria and Opa1 is required for efficient generation of memory CD8 T cells, presumably by facilitating this fusion.^26^ Surplus or damaged mitochondria can be removed in a process related to autophagy, called mitophagy. This ensures that mitochondria can efficiently produce ATP without increasing their production of damaging reactive oxygen species (ROS).

To increase transcription of metabolic genes associated with anabolic growth, cells can activate mammalian target of rapamycin (mTOR), a serine/threonine kinase that integrates a multitude of extracellular signals and intracellular cues and promotes glycolysis, growth and proliferation.^28^ Activation of mTOR controls the expression of a number of transcription factors, which will be described below. Of note, mTOR can be inhibited by AMP‐activated protein kinase (AMPK). AMPK senses the balance between AMP and ATP in the cell and drives catabolic metabolism when energy stores are depleted.^29^ It inhibits mTOR and other anabolic processes and also stimulates mitochondrial biogenesis, glucose and lipid uptake to restore energetic homoeostasis. As a result, both mTOR and AMPK form a key point of reciprocal regulation to balance catabolic and anabolic pathways during T cell activation and quiescence (Figure 1).

During OXPHOS, mitochondria can generate ROS, which include hydrogen peroxide (H_2_O_2_), the superoxide anion (O_2_^−^) and the hydroxyl radical (OH^–^). ROS can cause oxidative damage to cellular macromolecules but ROS is also a key signalling molecule for a number of physiological functions, including proliferation, cellular defence mechanisms, signal transduction and gene expression^30^ (Figure 1). For example, optimal T cell activation requires a burst of ROS.^31^ As a result, ROS production and redox balance must be tightly regulated by cellular antioxidant enzymes and modulators, such as GSH, to maintain proper signal transduction without compromising the integrity of the cell.

## Metabolism during naïve and memory T cell homoeostasis

During normal physiology, naïve and memory T cells must respond to homoeostatic cues that permit survival for prolonged periods and cellular metabolism can support this survival.

Naïve T cells are maintained in the periphery by several survival signals: tonic TCR signalling (a low‐level signal triggered by engagement of the TCR with self‐MHC), sphingosine‐1‐phosphate signalling and signalling from the common γ (γc) chain cytokine, interleukin (IL)‐7.^32^, ^33^, ^34^ During homoeostasis, naïve T cells are largely quiescent and have relatively low energy demands, which they meet through the use of OXPHOS, fuelled by glucose, amino acids and fatty acids.^19^ Glucose seems to be a critical substrate for OXPHOS in naïve T cells as both tonic TCR and IL‐7 signalling can promote Glut1 expression on T cells and inhibition of glycolysis promotes cell death.^35^, ^36^ However, more recent studies suggest that Glut1 expression is not required for peripheral survival of naïve T cells, suggesting that there is redundancy among glucose transporters.^37^ Regardless, TCR and IL‐7 signalling is required to mediate metabolic homoeostasis and survival in naïve T cells.^35^, ^38^

Memory T cells emerge after an immune response resolves, with their survival dependent on IL‐7 and/or IL‐15 signalling but independent of tonic TCR signalling.^32^, ^34^ These cells must survive in a quiescent state for very long periods and it was initially thought that memory T cells reverted to predominantly using FAO and OXPHOS to support this survival. However, when activated T cells were forced to sustain glycolysis through the continued expression of the hypoxia‐inducible factor 1α (HIF1α), memory cell populations still developed, although effector memory T (T_EM_) cells predominated rather than central memory T (T_CM_) cells.^39^ Memory T cells must respond rapidly upon secondary encounter with their cognate antigen. To facilitate this rapid response, memory T cells engage the early shift towards aerobic glycolysis more rapidly than naïve cells, which is thought to support rapid production of cytokines such as IFNγ.^40^, ^41^ At present, the balance between survival and rapid responsiveness is thought to lead to a unique metabolic state in memory T cells. They use glucose‐derived substrate for FAS, which is an anabolic process that generates rapidly accessible energy stores. They can simultaneously catabolise these stores through the action of a lysosomal lipase to generate fatty acids for use in FAO.^42^ Memory T cells are also thought to maintain more mitochondria per cell with a fused morphology and denser cristae,^26^, ^41^, ^43^ driving up the spare respiratory capacity, which should support more rapid engagement of effector functions. Altogether, these metabolic changes impart a metabolically primed state on memory T cells that facilitates rapid responses to subsequent stimulation.

Nevertheless, there is active debate regarding certain aspects of memory T cell metabolism. Firstly, a number of studies that investigated the impact of FAO on memory T cell function used etomoxir, which is an inhibitor of CptIa, to interrogate this metabolic pathway.^42^, ^43^ More recently, it was highlighted that etomoxir is non‐specific at high doses and the dose commonly used to inhibit FAO also inhibited the ETC.^44^, ^45^ These studies also used a T cell‐specific knockout of CptIa to suggest that FAO contributes minimally to memory T cell metabolism. Currently, the impact of FAO on memory cell metabolism remains contentious as these more recent studies have highlighted the need for a modified interpretation of past work. Secondly, a number of studies defined memory T cells as either CD8 T cells differentiated *in vitro* with IL‐15, or CD44^+^ CD8 T cells from naïve or previously infected mice. While these cells have a ‘memory‐like’ phenotype, they contain substantial populations of semi‐differentiated antigen‐naïve cells that have proliferated in response to IL‐15. Memory‐like populations can be generated during lymphopenia and include CD44^+^ virtual memory cells that accumulate with age. High spare mitochondrial capacity has been observed in lymphopenia‐induced cells,^46^ and similar metabolic adaptations are seen with increasing age in CD44^+^ CD8 T cells.^47^ This suggests that high spare respiratory capacity is uncoupled from antigen experience in T cells. As a result, some metabolic features thought to be characteristic of conventional antigen‐experienced memory T cells may actually be associated with IL‐15 signalling, lymphopenia and ageing.

## Metabolism and TCR signalling during T cell activation

Upon activation, T cells proliferate at an incredibly high rate and differentiate into effector T cells. This transition requires not only a sudden increase in energy generation but also the uptake and generation of biomolecules for proliferation, effector functions and trafficking.^18^ Our current understanding is that this occurs in a step‐wise manner, as detailed below.

Immediately after initial TCR engagement, there is an early upreagulation of aerobic glycolysis. TCR signalling leads to activation of PDH kinase 1, which phosphorylates and inactivates PDH.^48^ Normally, PDH facilitates the import of pyruvate into the mitochondria, so inhibition of PDH drives engagement of aerobic glycolysis.^48^ This shift towards aerobic glycolysis promotes cytokine production through several post‐transcriptional mechanisms. Glyceraldehyde 3‐phosphate dehydrogenase (GAPDH) is a crucial enzyme within the glycolytic pathway which has been shown to bind the 3′ untranslated region (UTR) of IFNγ mRNA to prevent its translation.^49^ When aerobic glycolysis is engaged, GAPDH releases the mRNA and IFNγ production is enabled in T cells. Activation of GAPDH is also potentiated in times of stress, as high levels of acetate are generated during catabolic stress to acetylate GAPDH and enhance its activity, thereby promoting glycolysis and rapid IFNγ production.^50^ Lactate dehydrogenase (LDH) is another key enzyme for aerobic glycolysis. It was initially reported that LDH did not alter IFNγ protein expression through 3′UTR interactions.^51^ However, a more recent report has shown that LDH does bind to IFNγ, IL‐2 and TNF mRNA; this binding is reduced with TCR activation and LDH may thereby provide an additional mechanism of control for IFNγ expression.^48^ Of note, these rapid, post‐transcriptional mechanisms are critical for rapid cytokine production by T cells, and they can prime the cell for more durable reprogramming and transcriptional changes. For example, LDH activity can reinforce IFNγ transcription by increasing the cellular concentration of acetyl‐CoA to increase histone acetylation and promoter accessibility at the *Ifng* gene locus.^51^

**Figure 2:**
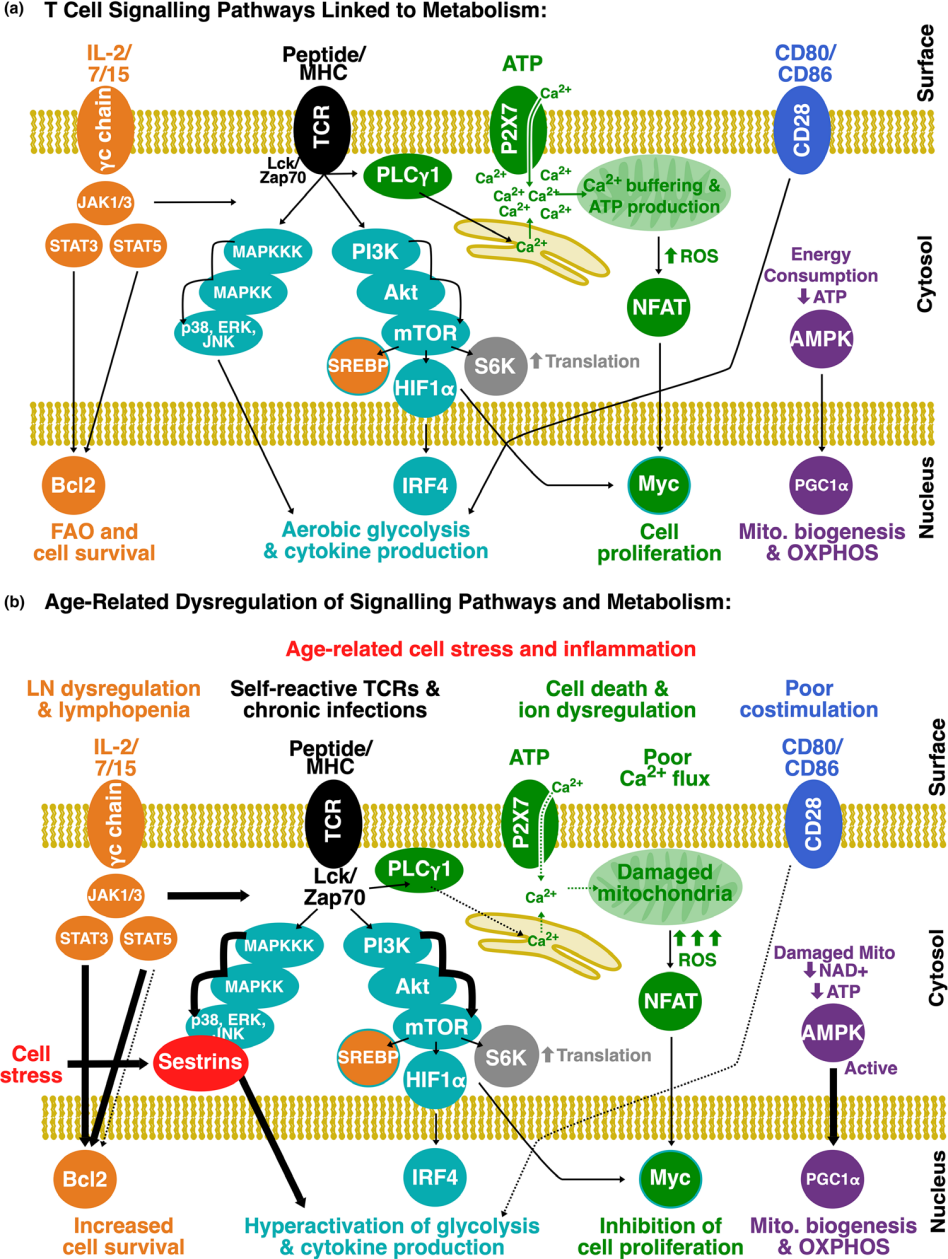
Summary of signalling pathways that regulate metabolism in T cells and how these pathways may change with age. **(a)** IL‐2, ‐7 or ‐15 signalling drives JAK/STAT signalling that can promote fatty acid oxidation and cell survival and augment T cell receptor (TCR)‐driven signalling pathways. TCR‐driven signalling drives MAPK, PI3K/Akt/mTOR and Ca^2+^ flux. MAPKs augment glycolysis, mTOR drives a host of transcription factors to promote cell division and aerobic glycolysis and Ca^2+^ flux with ROS promotes NFAT translocation and Myc‐mediated proliferation. Costimulatory signals mediated by CD28 augment glycolysis but also permit metabolic flexibility. Activation of AMPK by a reduction in cellular ATP levels results in mitochondrial biogenesis and an increase in oxidative phosphorylation. **(b)** Age‐related stress and inflammatory signals shift the balance of these signals in a T cell in the steady state and in response to infection. Lymph node dysregulation and decreased IL‐7 signalling leads to a loss of naïve T cells but modest lymphopenia may increase γc chain cytokine signalling in remaining T cells. Self‐reactive TCRs and chronic infections may increase basal TCR‐driven signalling and cell stress can drive sestrin activation to hyperphophorylate MAPKs. Ca^2+^ flux is impaired, with damage to mitochondria potentially playing a role in diminished ability to buffer local Ca^2+^ concentrations, undermining NFAT activation, Myc transcription and cell proliferation. AMPK is hyperactivated as a result of energy stress or nutrient sensing restrictions leading to increased mitochondrial biogenesis. The net effects of this dysregulated signalling are increased cell survival but inhibition of TCR‐driven proliferation, basal activation of glycolysis and mitochondrial biogenesis.

While early events engage aerobic glycolysis, this is followed by a substantial lag period, after TCR engagement but before a T cell initially divides, when a number of pathways downstream of TCR signalling mediate more durable transcriptional and metabolic shifts. These pathways include (1) calcium flux, (2) phosphinositide‐3 kinase (PI3K)‐Akt‐mTOR signalling and (3) mitogen‐activated protein kinase (MAPK) signalling (Figure 2a).

Calcium flux in T cells is precipitated by release of Ca^2+^ stores from the endoplasmic reticulum followed by an influx of extracellular Ca^2+^ (Figure 2a).^52^ Ca^2+^ influx leads to the activation of calcineurin, which phosphorylates the NFAT transcription factor to permit its translocation into the nucleus.^31^, ^53^ NFAT translocation drives the expression of Myc and IL‐2. Myc is a transcription factor that controls a variety of genes linked to proliferation and metabolic reprogramming,^54^ and IL‐2 can deliver autocrine signalling to support proliferation. Ca^2+^ influx is thereby crucial for proliferation of T cells, but NFAT also requires a burst of ROS from the mitochondria to trigger optimal signalling.^31^ Of note, excessive ROS or a lack of buffering from GSH can lead to inhibition of NFAT activation to limit T cell proliferation and other functions.^53^ This illustrates that metabolic regulation must delicately balance the redox state of activated T cells for optimal TCR‐mediated signalling.

Mitochondria are also essential for buffering the increase in intracellular Ca^2+^ (Figure 2a). They relocalise to the immune synapse where they can take up cytosolic Ca^2+^ to buffer local concentrations and sustain influx. This uptake of Ca^2+^ by mitochondria regulates the activity of enzymes within the TCA cycle and ETC to potentiate OXPHOS^55^. The relocalisation of mitochondria also promotes release of ATP into the immune synapse, which can act on ATP‐gated Ca^2+^ channels (extracellular purinergic receptors, such as PX27) to further sustain Ca^2+^ influx from the extracellular space, along with other Ca^2+^ channels.

T cell receptor signalling also leads to PI3K‐Akt‐mTOR signalling (Figure 2a). Activated Akt can phosphorylate intracellular reserves of Glut1 and promote its trafficking to the cell surface to promote glucose uptake.^56^, ^57^ mTOR is a pivotal integration point in cellular signalling, downstream of growth factors and sensors of nutrient availability.^58^ In response to these signals, mTOR coordinates the activation or expression of a number of different transcription factors that promote T cell activation and metabolic remodelling. These include Myc, sterol regulatory element binding proteins (SREBP1 and SREBP2; drive FAS and cholesterol synthesis), HIF1α (drives sustained aerobic glycolysis) and interferon regulatory factor 4 (augments aerobic glycolysis and integrates TCR signalling strength).^54^, ^59^, ^60^, ^61^ mTOR also promotes activation of ribosomal protein S6 kinase (S6K), which is a component of the 40S ribosome, to augment protein translation and build biomass. As a result, mTOR acts as a gatekeeper, by controlling cell size, entry into cell cycle and protein production, to ensure proliferation is limited to nutrient conditions that support proliferation.

T cell receptor signalling also drives activation of MAPK signalling cascades, with activation of p38, extracellular signal–regulated kinases 1/2 (ERK1/2) and c‐Jun N‐terminal kinase (JNK; Figure 2a). Again, these MAPKs integrate a number of TCR, cytokine and stress signals and modulate a wide range of targets to promote T cell proliferation. MAPK signalling has a substantial impact of engagement of glycolysis, as inhibition of ERK 1/2 and, to a lesser extent, p38 can limit glycolysis and cell proliferation after TCR stimulation.^62^

Of note, while there has been much focus on the upregulation of aerobic glycolysis after TCR engagement, OXPHOS is similarly upregulated and it remains a crucial metabolic pathway for T cells after activation. Indeed, T cell activation drives substantial mitochondrial biogenesis to meet the increased energy demands of effector T cells, and if OXPHOS is blocked, T cell proliferation is inhibited.^49^ The newly generated mitochondria are also qualitatively distinct, with substantial upregulation of enzymes related to one‐carbon metabolism.^25^ Overall, it is clear that upregulation of both the glycolytic and OXPHOS pathways is critical for effective T cell activation.

## Costimulatory or cytokine signalling

Costimulatory or cytokine signalling can augment transcriptional shifts in activated T cells and qualitatively alter metabolic outcomes (Figure 2a). As an example, CD28 signalling synergises with TCR signalling to further potentiate Akt activation and promote surface expression of Glut1.^63^ CD28 signalling also supports metabolic flexibility, so when glucose is limited, a cell that has received CD28 signals can switch to supplement energy production with OXPHOS^63^ and cells can undergo transition to a memory population and FAO usage more readily.^64^

Cytokines that utilise the γc chain, such as IL‐2, ‐7 and ‐15, can crosstalk with cellular metabolism in a number of ways (Figure 2a). In general, the γc chain cytokines support survival and maintain biomass^38^ but they also cause transcriptional changes that modify nutrient uptake. For example, IL‐7 signalling leads to upregulation of aquaporin 9 in memory cells, but not naïve T cells, to import glycerol and store it for FAO.^65^ The γc chain cytokines also trigger signalling through the JAK/STAT3/5 pathways to promote cell survival by driving expression of anti‐apoptotic Bcl‐2 family members, such as Bcl‐2.^36^, ^38^ More broadly, cytokines are critical for guiding differentiation of specific CD4 T cell helper subsets. Cytokine‐driven signalling appears to coordinate differentiation at least in part through mTOR signalling, as lack of mTOR undermines the differentiation of Th1, Th2 and Th17 cells.^66^

## Nutrient sensing in T cells

T cells in circulation can access an abundance of nutrients and substrate for metabolic pathways, but substrate usage across T cell differentiation states can also be controlled by limiting the expression of transporters to restrict nutrient uptake. Resting naïve T cells rely on OXPHOS driven by glucose and resting memory T cells can also utilise glucose for FAS coupled to FAO, but other substrates are not drawn upon to a significant extent. These resting T cells express modest levels of the glucose transporter, Glut1, as a result of homoeostatic signalling to support uptake of substrate for their respective metabolic programmes. In contrast, T cells upregulate a number of nutrient transporters after activation and rely on sensing of nutrient availability to regulate their differentiation and function.

T cell activation requires glucose for cell growth, proliferation and cytokine production. TCR signalling drives Akt activation to increase trafficking and cell surface expression of Glut1 and to support glycolysis.^57^ Similarly, T cell activation requires glutamine for efficient CD28‐dependent T cell activation, proliferation and cytokine production. TCR and costimulatory signalling triggers ERK phosphorylation to drive upregulation of a glutamine transporters, such as CD98, SNAT1 and SNAT2, effectively linking TCR‐mediated activation with glutaminolysis.^21^ Moreover, CD98 facilitates the import of a range of large neutral amino acids, such as leucine and methionine, which augment T cell activation.^67^, ^68^ CD98 is therefore a pivotal amino acid transporter in activated T cells and it may represent a therapeutic target for ageing, where partial inhibition could reduce chronic T cell activation.

In addition to controlled expression of transporters, access to metabolic substrates may become limited in certain environments, such as in the lymph node with large numbers of proliferating T cells or in the tumor microenvironment with large numbers of proliferating malignant cells. Decreased access to glucose,^63^ glutamine,^54^ l‐arginine,^69^ cholesterol,^61^ methionine,^67^ folate,^70^ serine, glycine and formate^24^, ^25^ can all limit T cell activation. The microbiome can even influence the metabolic profile of cells. Normal commensal flora ferment dietary fibre to produce short‐chain fatty acids like butyrate, which strongly promotes FAO, OXPHOS and memory T cell development.^71^ While many nutrients are imported, it should be noted that autophagy can also be a source of macromolecules and is essential for the resolution of an immune response, to establish a memory cell population.^72^

## Global mechanisms of ageing

**Figure 3:**
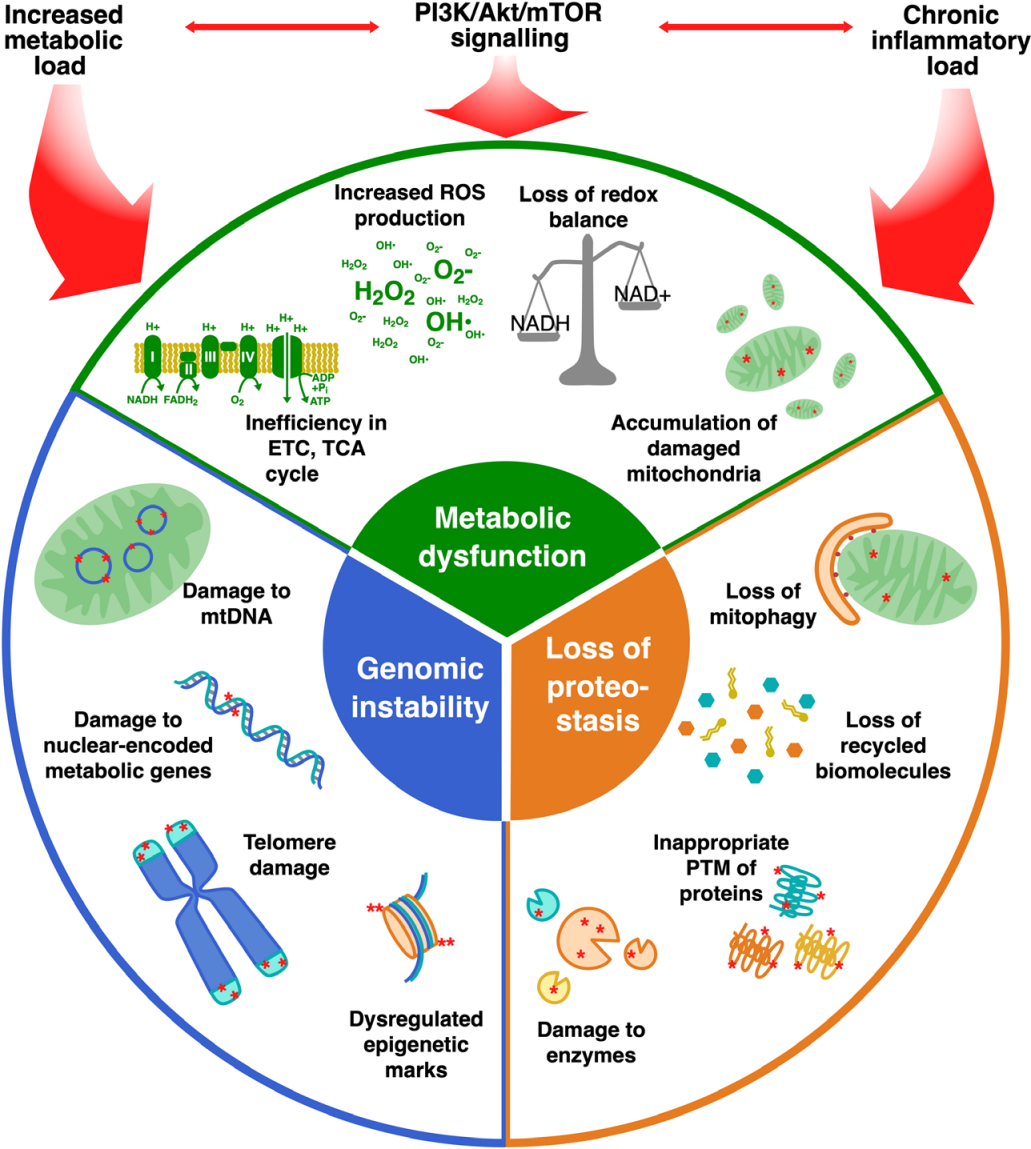
Selected hallmarks and mechanisms of metabolism‐mediated cellular ageing. Key hallmarks include genomic instability, loss of proteostasis and metabolic dysfunction. Genomic instability leads to (1) damage to mtDNA, (2) mutations in metabolic genes, (3) telomere erosion or (4) dysregulation of epigenetic marks. Loss of proteostasis leads to (1) damage to enzymes, (2) inappropriate post‐translational modifications, (3) a deficit of recycled biomolecules and (4) a loss of mitophagy. Metabolic dysfunction leads to (1) an accumulation of dysfunctional mitochondria, (2) loss of redox balance in the cell, (3) increased ROS production and (4) inefficiencies in metabolic pathways. These outcomes can be accelerated with increased metabolic load, increased mTOR signalling or chronic inflammation, all of which are inter‐related mechanisms of cellular ageing.

Before discussing the impact of age on T cell metabolism specifically, we will briefly discuss broader features of cellular ageing. Ageing cells exhibit a number of very similar hallmarks regardless of cell type. These include loss of genomic stability, loss of proteostasis and metabolic dysfunction (reviewed by López‐Otín *et al*.^73^). Genomic instability may alter metabolism through (1) mutations in mitochondrial genes or nuclear‐encoded metabolic genes,^74^ (2) telomere erosion‐initiated or DNA damage‐initiated activation of the p53‐regulated DNA damage repair response to inhibit mitochondrial biogenesis^75^ or (3) dysregulation of metabolic gene expression through changes in DNA methylation, histone modifications or availability of transcription factors and other regulatory mechanisms^76^, ^77^ (Figure 3). Loss of autophagy and proteostasis appears to impact metabolism,^78^, ^79^ and it may do this through mechanisms such as (1) accumulated damage to metabolic enzymes, (2) inappropriate post‐translational modifications to enzymes, (3) a loss of recycled biomolecules for catabolism and (4) a decrease in mitophagy to turnover damaged mitochondria (Figure 3). Metabolic dysfunction is likely to be the net outcome of many mechanisms, including those described above, which can result in (1) an accumulation of dysfunctional mitochondria with damage to mtDNA, (2) increased ROS production, which may cause oxidative damage if in excess,^80^ (3) decreased levels of the redox cofactor, NAD^+^, in the cell^81^ and (4) inefficiencies in metabolic pathways caused by damaged DNA and proteins (Figure 3). Indeed, for many years, the mitochondrial (and free radical) theory of ageing suggested that mitochondrial dysfunction was the central driver of cellular ageing.^80^ While the concept that mitochondria is the sole driver of ageing has fallen out of favor, mitochondria clearly become dysfunctional with regard to key T cell functions, such as calcium signalling.^82^ This illustrates that age‐related mitochondrial dysfunction can underlie age‐related T cell dysfunction.

The accumulation of these ageing hallmarks can be impacted by an individual's rates of metabolism (Figure 3). As an example, caloric restriction can reduce metabolic rates, reduce age‐related dysfunction in a number of cell types and increase lifespan.^13^ The impact of metabolism on ageing is mediated at least in part by the PI3K‐Akt‐mTOR signalling pathway, as inhibition of mTOR can significantly extend lifespan in multiple different species, including yeast, nematodes and mice.^83^ mTOR is therefore a highly conserved regulator of both metabolism and longevity.

The accumulation of these ageing hallmarks can also be impacted by chronic inflammation (Figure 3), particularly in immune cells. Heightened expression of cytokines, such as TNF, IL‐6, interferons (IFN) and IL‐1β, are seen in older individuals and increased inflammation is associated with premature ageing phenotypes, in a process called inflammaging.^84^ This increased inflammatory signalling can increase both PI3K/Akt/mTOR signalling and metabolic rates.^85^ During normal biological ageing, chronic inflammation may have a number of sources. First, chronic infections such as CMV and Epstein‐Barr virus (EBV) can stimulate the production of these cytokines.^86^ Secondly, increased adiposity is observed with age and adipose tissue can be a major source of TNF, IL‐6, IFNγ and IL‐1β, as well as indicative of broader metabolic dysfunction.^87^ Thirdly, ageing of the gut can cause dysbiosis and leakiness of bacterial products to drive chronic immune activation.^88^ Finally, DNA damage and loss of proteostasis drive the development of senescent cells throughout the body in older individuals. These senescent cells can exhibit a senescence‐associated secretory phenotype (SASP), which leads to the production of highly inflammatory cytokines.^89^ Given the inter‐relatedness of ageing processes (Figure 3), any age‐related deficit in T cells is likely to be the cumulative outcome of a number of age‐related mechanisms.

## Impact of ageing on T cell signalling pathways

In aged T cells, evidence of these ageing hallmarks can be readily observed. T cells recovered from aged individuals have shorter telomeres in both their naïve and memory populations^90^ and they exhibit increased expression of γH2Ax, which is indicative of the DNA damage repair response.^91^ T cells recovered from older individuals exhibit decreased basal level of autophagy,^92^ while T cells from the offspring of particularly long‐lived individuals have robust activation‐induced autophagic responses.^93^ Senescent T cell subsets and T cells from older individuals exhibit a number of mitochondrial and metabolic defects.^94^, ^95^, ^96^ Age‐related inflammation may exacerbate these phenotypes. For example, type I IFNs can block telomere repair in T cells,^97^ and chronic inflammation or infections are associated with premature immune ageing.^84^, ^85^ T cells are therefore clearly subject to general ageing mechanisms but there may also be T cell‐specific ageing mechanisms.

Aged T cells also exhibit dysregulation of a number of signalling pathways that are linked to metabolism. In resting T cells, homoeostatic signalling can be altered with age (Figure 2b). For example, IL‐7 signalling is known to decline in aged mice and humans,^3^ which is likely to undermine the metabolic fitness of naïve T cells. In contrast, aged mice and individuals that have lost T cells may enter a state of modest lymphopenia, which may increase γc signalling for the remaining T cells. Tonic TCR signalling may also change, as T cell subsets with more self‐reactive TCRs appear to accumulate with age.^5^, ^7^

After activation, a number of age‐related deficits have been described in T cell signalling pathways (Figure 2b). The impact of ageing on early events after TCR engagement is not well defined, but ageing clearly impacts on Ca^2+^ flux. As we get older, ion homoeostasis is often dysregulated, leading to blunted Ca^2+^ flux,^98^ and the structural and functional integrity of mitochondria can decline, leading to dysregulation of ROS. This would be predicted to dysregulate Ca^2+^ flux‐mediated signals, particularly NFAT signalling and undermine the ability of mitochondria to buffer and sustain Ca^2+^ signalling. The availability of costimulatory signals and cytokines can change markedly with age, which is likely to impact on the metabolic profile of T cells. Critically, both nutrient sensing and autophagy are known to be dysregulated with cellular ageing^73^ and in senescent T cells.^94^ Supplementation of age‐limited nutrients, such as formate and glycine,^96^ and augmenting autophagy through spermidine exposure^99^ can improve the functional capacity of aged T cells. This illustrates that improving the access to or recycling of biomolecules may influence the function of aged T cells.

However, the most dramatic age‐related difference is that resting aged T cells exhibit increased basal activation of PI3K/Akt/mTOR and MAPK signalling pathways (Figure 2b). Dominant activating mutations in PI3K cause T cell dysfunction and immunosenescence,^100^ so chronic activation of the PI3K/Akt/mTOR pathway is predicted to drive dysfunction. Consistent with this, chronic infections, such as HIV, can deliver sustained TCR and inflammatory cytokine signals to memory T cells. This leads to chronic PI3K/Akt/mTOR pathway activation and T cell dysfunction, as well as increased basal Glut1 expression and increased basal glycolytic activity.^85^ Aged senescent memory T cells also exhibit hyperphosphorylation of MAPK cascades,^91^ which appears to be precipitated by a cellular stress‐related response coordinated by a protein complex of MAPKs (p38, ERK, JNK) and stress‐induced proteins called sestrins. The sestrin‐MAPK complex inhibits responses in aged T cells, but proliferative capacity, Ca^2+^ flux and cytokine production could be restored with sestrin knockdown.^91^ Sestrins are thought to have anti‐ageing properties, with the capacity to suppress oxidative stress and regulate AMPK‐mTOR signalling,^101^ but sestrin‐mediated T cell dysfunction appears to occur independently of mTOR, through dysregulation of MAPK activation. Of note, senescent memory T cells that have increased basal MAPK activation also exhibit increased basal glycolytic rates.^47^, ^91^, ^94^ AMPK is also hyperactivated in senescent and aged T cells,^91^ possibly as a response to ATP deficit in the cell, which augments basal mitochondrial biogenesis. As a result, hyperactivation of PI3K/Akt/mTOR, MAPK and AMPK signalling pathways are hallmarks of T cell ageing, which manifest in an increased glycolytic rate and mitochondrial mass.

## Metabolic profiles in age‐associated T cell subsets

While direct analyses of metabolic changes in aged T cell subsets have been limited, several studies have defined the metabolic profile of T cells that accumulate with age, namely exhausted, terminally differentiated and T_EMRA_ cells.

T cells can become exhausted with sustained TCR signalling during chronic infections such as CMV and EBV. This can lead to the upregulation of PD‐1, which in turn has been shown to downregulate glycolytic metabolism.^102^ Aged T cell populations can contain higher proportions of exhausted T cells and this form of metabolic insufficiency is likely to be a key driver of age‐related T cell dysfunction.

Terminally differentiated memory CD8^+^CD28^−^ T cells are a dysfunctional population that is significantly enriched in older individuals. They exhibit defects in TCR‐mediated proliferation and are thought to drive many aspects of immune dysregulation in the elderly.^103^ CD8^+^CD28^−^ T cells exhibit marked downregulation of Sirtuin (SIRT)1, an NAD^+^‐dependent protein deacetylase that targets a number of transcription factors regulating metabolism and age‐related processes.^104^ Age‐related loss of SIRT1 is observed in many other ageing tissues and is known to decrease mitochondrial capacity and increase senescence in stem cells, to limit organismal lifespan.^105^ Jeng and colleagues found that lack of SIRT1 also led to an increase in glycolytic capacity and granzyme B production in resting, but not activated, CD8^+^CD28^−^ T cells.^104^

Finally, T_EMRA_ cells are non‐proliferative T cells that accumulate with age and exhibit features of senescence, in that they exhibit markers of DNA damage and cell cycle arrest.^94^, ^106^ T_EMRA_ cells have low numbers of mitochondria, and they do not efficiently upregulate glycolysis or OXPHOS compared to other T cell subsets after TCR activation, although they have high ROS production.^94^ Proliferation was significantly improved in T_EMRA_ cells by inhibiting p38 MAPK signalling,^94^ which was associated with increased numbers of mitochondria and increased autophagy.^94^ Collectively, while these studies did not directly analyse aged T cells, they indicate that dysfunction in T cell populations can be driven by enrichment of exhausted, terminally differentiated or T_EMRA_ cells in the elderly with dysfunction caused, at least in part, by metabolic programming.

Ageing can augment accumulation of specific subsets by promoting their selective proliferation or differentiation, and also the selective survival of cell subsets. Indeed, a key feature of aged T cells is their augmented survival capacity.^47^, ^107^ Even non‐senescent memory CD8 T cells from aged mice and humans exhibit a survival advantage *in vitro* after induction of apoptosis.^108^, ^109^ Studies have attributed much of the preferential survival capacity of aged T cells to heightened Bcl‐2 expression.^47^, ^107^ However, increased production of antioxidants such as GSH has been shown to protect aged T cells against oxidative stress and loss of mitochondrial membrane potential,^108^ thereby promoting cell survival.

## Perturbations in aged T cell metabolism

In addition to shifts in the composition of T cell populations, ageing can directly alter the metabolism of specific T cell subsets, such as naïve T cells and conventional memory T cell subsets.

With regard to naïve T cells, a recent study of epigenetic changes in unstimulated human naive CD8 T cells demonstrated an increase in mitochondrial mass but, somewhat counterintuitively, a loss of mitochondrial respiratory capacity in aged cells.^95^ The loss of respiratory capacity appeared to be driven by diminished expression of ETC genes, which in turn was attributed to a reduced capacity for NRF1 to maintain openness of ETC gene promoters. This illustrates that aged cells can accumulate mitochondrial mass but mitochondrial quality may not support efficient respiration. The impact of a qualitative deficit in mitochondria was highlighted during a study with young and aged murine naive CD4 T cells.^96^ Aged CD4 T cells exhibited a defect in both glycolysis and OXPHOS after TCR‐driven activation, but proteomic analysis showed no change in the mitochondria from young and aged cells in the induction of enzymes involved in TCA cycle, ETC or FAO. However, there was a striking deficiency in aged cells in the upregulation of enzymes required for one‐carbon metabolism,^96^ which was previously shown to be critical for naïve T cell activation.^25^

With regard to memory T cells, increased age led to moderately elevated expression of mTOR and phosphorylated Akt and ribosomal protein S6K in resting memory CD8 T cells.^47^ Increased S6K phosphorylation also correlated with an increase in size and granularity of aged compared to young memory T cells.^47^ While mitochondrial mass was unchanged, resting aged memory CD8 T cells exhibited increased glucose utilisation, with elevated Glut1 and insulin receptor expression and 2‐NDBG uptake in the steady state. Similarly, an age‐associated increase in glycolysis in resting memory T cells was observed in T_EMRA_ cells,^94^ which selectively accumulate in humans with age, and in functionally exhausted T cells responding to chronic viral infection.^110^ In activated human memory CD4 T cells, no defect in glycolysis or OXPHOS was evident upon activation.^111^ In fact, OXPHOS was modestly increased in aged T cells, but they generated disproportionately more ROS and more intra‐ and extracellular ATP, to trigger P2X7 and promote Ca^2+^ influx.

These studies represent the first definitive demonstrations of age‐related metabolic alterations in the T cell subsets, but whether such changes are a cause of age‐related T cell dysfunction or a by‐product of the ageing process remain unclear. It is also unresolved as to whether age‐related metabolic shifts primarily affect the homoeostatic survival of T cells or the capacity to become activated and acquire effector functions. It may be that the observed metabolic alterations are adaptations that promote survival of aged T cells, but this comes at the cost of critical T cell functions such as proliferation.

## Metabolic interventions to improve T cell immunity in the elderly

Given the impact of metabolism on both ageing and T cell function, a number of studies have attempted to manipulate metabolism to prevent exhaustion/senescence or augment aged T cell function.^112^, ^113^ As previously outlined, exercise and caloric restriction are interventions that offer both general anti‐ageing effects and T cell‐specific effects, with increased longevity, function and thymic output,^12^, ^13^, ^14^ but pharmacological interventions are also being explored.

A common characteristic of aged dysfunctional T cells is heightened steady‐state glycolytic metabolism. Blocking glycolytic metabolism in young CD8 T cells has been shown to promote generation of long‐lived, functional memory populations, while enforcing glycolysis drives CD8 T cells towards a terminally differentiated state.^39^, ^114^ One way to prevent accumulation of terminally differentiated senescent T cells and promote survival of functional memory T cells in advanced age could include inhibitors or regulators of glycolysis, such as 2‐deoxyglucose, which directly inhibits glycolysis^114^ or metformin, which indirectly decreases glycolysis via activation of AMPK.^112^

Metformin is of particular interest as a metabolic modulator in ageing. It has a robust safety profile, having been used for over 60 years as a first‐line medication to promote glycaemic control in type 2 diabetes. Moreover, metformin has been proposed to target mechanisms related to ageing through a number of modes of action, which include activating AMPK, inhibiting complex I in the ETC, reducing ROS, reducing insulin‐like growth factor‐1 signalling and inhibiting mTOR (reviewed by Barzilai *et al*.^115^). While the precise mechanisms responsible for the anti‐ageing effect of metformin are not well defined, metformin treatment reduced apoptosis and promoted memory CD8 T cell formation in young adult mice, to improve immunity against subsequent virus infection and tumor challenge.^116^ Metformin treatment has also been shown to suppress immune responses, namely by impairing Th1/Th17 differentiation and promoting Treg differentiation,^117^ providing benefit in some autoimmune models. Metformin may be beneficial for the generation of T cell memory, but it inhibits proliferation, cytotoxicity and survival of chimeric antigen receptor (CAR) T cell during production, via its effect on AMPK.^118^ Critically, while metformin may promote the development of T cell immunity in young individuals, the effects of metformin in aged individuals on T cells remains to be specifically assessed.

The mTOR pathway is another promising target for mitigating cellular ageing. mTOR can be inhibited by rapamycin treatment, which has been shown to reverse age‐related immunosenescence in aged mice, in part by restoring both the self‐renewal and haematopoietic capacity of aged haematopoetic stem cells.^119^ In two studies, low‐dose mTOR inhibitors were administered to elderly individuals for 6 weeks and prior to influenza vaccination. This led to a markedly fewer infections and a reduction in PD‐1 expression on circulating CD4 and CD8 T cells.^120^, ^121^ It is not clear whether this is mediated by direct inhibition of mTOR in T cells, but this finding is supported by mouse and non‐human primate models. In these models, mTOR inhibition caused little change in the effector T cell response magnitude, but significantly enhanced memory CD8 T cell differentiation after challenge with a range of pathogens or vaccines.^122^, ^123^

Supplementation of redox cofactors and nutrients in aged T cells may also improve function. NAD^+^ supplementation has been widely documented to mitigate age‐related biological decline and to promote mitochondrial function in a number of other cell types.^105^, ^124^ As previously mentioned, CD8^+^CD28^−^ T cells in humans exhibit reduced SIRT1 levels, which is predicted to undermine mitochondrial capacity and increase cellular senescence.^104^ NAD^+^ supplementation has been shown to recover mitochondrial and cellular function in aged, senescent mouse stem cells via a SIRT1‐dependent mechanism,^105^ therefore boosting SIRT1 activity via NAD^+^ supplementation may reverse T cell dysfunction.

A potential T cell‐specific target for functional recovery is the sestrin proteins. Lanna and colleagues showed that proliferation in senescent CD4^+^CD27^−^CD28^−^ T cells can be increased by knockdown of sestrins.^91^ While sestrins promote an ageing phenotype in T cells, it should be noted that sestrins also protect against a number of metabolic diseases that increase in incidence with ageing, such as diabetes, obesity, cancer and atherosclerosis.^101^ As a result, systemic sestrin inhibition would not be an optimal approach but *ex vivo* conditioning of aged T cells prior to adoptive cellular immunotherapy may be beneficial.

While the interventions listed above are promising, they are likely to modulate the metabolism of both T cells and other cell types. This may be desirable if the aim is to mitigate a common mechanism of ageing, but if the aim is to modify a given metabolic process specifically in T cells, then more targeted approaches need to be developed. There is substantial heterogeneity across T cell subsets in their response to ageing,^107^ and metabolic interventions that aim to restore T cell function may have to target the specific metabolic dysfunction in a specific subset: T_N_ cells, T_EFF_ cells, T_EM_ or T_CM_ cells, T_VM_ cells, exhausted cells, terminally differentiated cells and T_EMRA_ cells. One approach is to isolate specific T cell subsets for use in cell‐based therapies, such as CAR T cell therapy, with the inclusion of metabolic drugs during *in vitro* culture. Another approach, developed predominantly in the context of anti‐tumor immunity, is to use *in vivo* targeting strategies such as antibody‐targeted delivery or transporter‐facilitated uptake of metabolic inhibitors.^113^

Of note, some potential metabolic interventions, such as mTOR inhibitors, aim to augment memory formation while inhibiting the effector response. Such interventions can be administered concurrently with vaccines to augment vaccine‐induced responses, but there was some concern that long‐term administration of mTOR inhibitors could inhibit infection‐induced responses.^125^ Reassuringly, a recent study suggests that extended treatment with rapamycin analogues provides anti‐ageing effects as well as enhanced protection against infections.^121^ This approach highlights that, during future development of interventions for age‐related T cell metabolic dysfunction, it will be important to define both direct and indirect impacts of interventions on the T cells themselves.

## Summary

In summary, T cells rely on a number of signalling pathways and downstream engagement of metabolic pathways to both maintain homoeostasis and respond to TCR and cytokine stimulation during activation. Ageing can perturb these pathways, most notably by leading to basal hyperactivation of signalling pathways in resting T cells, which can lead to increased basal glycolytic rates. While currently there is often not sufficient information to ascribe a specific age‐related metabolic profile to a specific age‐related functional deficit in T cells, this area of research is building. As a result, interventions that target T cell metabolic dysfunction or signalling hold great promise to remedy age‐related deficits, by improving T cell retention and function.

## Conflict of interest

The authors declare no conflict of interest.

## References

[1] Goronzy JJ , Weyand CMW . Mechanisms underlying T cell ageing. Nat Rev Immunol 2019; 19: 573–583. 31186548 10.1038/s41577-019-0180-1 PMC7584388

[2] Nikolich‐Žugich J . The twilight of immunity: emerging concepts in aging of the immune system. Nat Immunol 2018; 19: 10–19. 29242543 10.1038/s41590-017-0006-x

[3] Becklund BR , Purton JF , Ramsey C *et al* The aged lymphoid tissue environment fails to support naïve T cell homeostasis. Sci Rep 2016; 6. 10.1038/srep30842 PMC4969611 27480406

[4] Goronzy JJ , Fang F , Cavanagh MM *et al* Naive T cell maintenance and function in human aging. J Immunol 2015; 194: 4073–4080. 25888703 10.4049/jimmunol.1500046 PMC4452284

[5] Quinn KM , Zaloumis SG , Cukalac T *et al* Heightened self‐reactivity associated with selective survival, but not expansion, of naïve virus‐specific CD8^+^ T cells in aged mice. Proc Natl Acad Sci USA 2016; 113: 1333–1338. 26787864 10.1073/pnas.1525167113 PMC4747731

[6] Drobek A , Moudra A , Mueller D *et al* Strong homeostatic TCR signals induce formation of self‐tolerant virtual memory CD8 T cells. EMBO 2018; 37: e98518. 10.15252/embj.201798518 PMC6043851 29752423

[7] Rudd BD , Venturi V , Li G *et al* Nonrandom attrition of the naive CD8^+^ T‐cell pool with aging governed by T‐cell receptor:pMHC interactions. Proc Natl Acad Sci USA 2011; 108: 13694–13699. 21813761 10.1073/pnas.1107594108 PMC3158207

[8] Vescovini R , Biasini C , Fagnoni FF *et al* Massive load of functional effector CD4^+^ and CD8^+^ T cells against cytomegalovirus in very old subjects. J Immunol 2007; 179: 4283–4291. 17785869 10.4049/jimmunol.179.6.4283

[9] Briceno O , Lissina A , Wanke K *et al* Reduced naïve CD8^+^ T‐cell priming efficacy in elderly adults. Aging Cell 2016; 15: 14–21. 26472076 10.1111/acel.12384 PMC4717282

[10] Schulz AR , Maelzer JN , Domingo C *et al* Low thymic activity and dendritic cell numbers are associated with the immune response to primary viral infection in elderly humans. J Immunol 2015; 195: 4699–4711. 26459351 10.4049/jimmunol.1500598

[11] Simpson RJ , Lowder TW , Spielmann G *et al* Exercise and the aging immune system. Ageing Res Rev 2012; 11: 404–420. 22465452 10.1016/j.arr.2012.03.003

[12] Nieman DC , Henson DA , Gusewitch G *et al* Physical activity and immune function in elderly women. Med Sci Sports Exerc 1993; 25: 823–831. 8350705 10.1249/00005768-199307000-00011

[13] Messaoudi I , Warner J , Fischer M *et al* Delay of T cell senescence by caloric restriction in aged long‐lived nonhuman primates. Proc Natl Acad Sci USA 2006; 103: 19448–19453. 17159149 10.1073/pnas.0606661103 PMC1748246

[14] Duggal NA , Pollock RD , Lazarus NR *et al* Major features of immunesenescence, including reduced thymic output, are ameliorated by high levels of physical activity in adulthood. Aging Cell 2018; 17: e12750. 10.1111/acel.12750 PMC5847865 29517845

[15] Almeida L , Lochner M , Berod L *et al* Metabolic pathways in T cell activation and lineage differentiation. Sem Immunol 2016; 28: 514–524. 10.1016/j.smim.2016.10.009 27825556

[16] Wang R , Green DR . Metabolic reprogramming and metabolic dependency in T cells. Immunol Rev 2012; 249: 14–26. 22889212 10.1111/j.1600-065X.2012.01155.x PMC3422760

[17] Ron‐Harel N , Sharpe AH , Haigis MC . Mitochondrial metabolism in T cell activation and senescence: a mini‐review. Gerontology 2015; 61: 131–138. 25402204 10.1159/000362502

[18] Palmer CS , Ostrowski M , Balderson B *et al* Glucose metabolism regulates T cell activation, differentiation, and functions. Front Immunol 2015; 6: 1. 25657648 10.3389/fimmu.2015.00001 PMC4302982

[19] Guppy M , Greiner E , Brand K . The role of the Crabtree effect and an endogenous fuel in the energy metabolism of resting and proliferating thymocytes. Eur J Biochem 1993; 212: 95–99. 8444168 10.1111/j.1432-1033.1993.tb17637.x

[20] Raud B , McGuire PJ , Jones RG *et al* Fatty acid metabolism in CD8^+^ T cell memory: Challenging current concepts. Immunol Rev 2018; 283: 213–231. 29664569 10.1111/imr.12655 PMC6691976

[21] Carr EL , Kelman A , Wu GS *et al* Glutamine uptake and metabolism are coordinately regulated by ERK/MAPK during T lymphocyte activation. J Immunol 2010; 185: 1037–1044. 20554958 10.4049/jimmunol.0903586 PMC2897897

[22] Wang T , Marquardt C , Foker J . Aerobic glycolysis during lymphocyte proliferation. Nature 1976; 261: 702–705. 934318 10.1038/261702a0

[23] Rosenzweig A , Blenis J , Gomes AP . Beyond the Warburg effect: How do cancer cells regulate one‐carbon metabolism? Front Cell Dev Biol 2018; 6: 90. 30159313 10.3389/fcell.2018.00090 PMC6103474

[24] Ma EH , Bantug G , Griss T *et al* Serine is an essential metabolite for effector T cell expansion. Cell Metab 2017; 25: 345–357. 28111214 10.1016/j.cmet.2016.12.011

[25] Ron‐Harel N , Santos D , Ghergurovich JM *et al* Mitochondrial biogenesis and proteome remodeling promote one‐carbon metabolism for T cell activation. Cell Metab 2016; 24: 104–117. 27411012 10.1016/j.cmet.2016.06.007 PMC5330619

[26] Buck MD , O’Sullivan D , Klein Geltink RI *et al* Mitochondrial dynamics controls T cell fate through metabolic programming. Cell 2016; 166: 63–76. 27293185 10.1016/j.cell.2016.05.035 PMC4974356

[27] Chen H , Chan DC . Physiological functions of mitochondrial fusion. Ann N Y Acad Sci 2010; 1202: 21–25. 10.1111/j.1749-6632.2010.05615.x 20649534

[28] Morita M , Gravel S‐P , Chénard V *et al* mTORC1 controls mitochondrial activity and biogenesis through 4E‐BP‐dependent translational regulation. Cell Metab 2013; 18: 698–711. 24206664 10.1016/j.cmet.2013.10.001

[29] Blagih J , Coulombe F , Vincent EE *et al* The energy sensor AMPK regulates T cell metabolic adaptation and effector responses *in vivo* . Immunity 2015; 42: 41–54. 25607458 10.1016/j.immuni.2014.12.030

[30] Klein Geltink RI , O’Sullivan D , Pearce EL . Caught in the cROSsfire: GSH controls T cell metabolic reprogramming. Immunity 2017; 46: 525–527. 28423332 10.1016/j.immuni.2017.03.022 PMC5580393

[31] Sena LA , Li S , Jairaman A *et al* Mitochondria are required for antigen‐specific T cell activation through reactive oxygen species signaling. Immunity 2013; 38: 225–236. 23415911 10.1016/j.immuni.2012.10.020 PMC3582741

[32] Tanchot C , Lemonnier FA , Pérarnau B *et al* Differential requirements for survival and proliferation of CD8 naïve or memory T cells. Science 1997; 276: 2057–2062. 9197272 10.1126/science.276.5321.2057

[33] Mendoza A , Fang V , Chen C *et al* Lymphatic endothelial S1P promotes mitochondrial function and survival in naive T cells. Nature 2017; 546: 158–161. 28538737 10.1038/nature22352 PMC5683179

[34] Murali‐Krishna K , Lau LL , Sambhara S *et al* Persistence of memory CD8 T cells in MHC class I‐deficient mice. Science 1999; 286: 1377–1381. 10558996 10.1126/science.286.5443.1377

[35] Rathmell JC , Vander Heiden MG *et al* In the absence of extrinsic signals, nutrient utilization by lymphocytes is insufficient to maintain either cell size or viability. Mol Cell 2000; 6: 683–692. 11030347 10.1016/s1097-2765(00)00066-6

[36] Wofford JA , Wieman HL , Jacobs SR *et al* IL‐7 promotes Glut1 trafficking and glucose uptake via STAT5‐mediated activation of Akt to support T‐cell survival. Blood 2008; 111: 2101–2111. 18042802 10.1182/blood-2007-06-096297 PMC2234050

[37] Macintyre AN , Gerriets VA , Nichols AG *et al* The glucose transporter Glut1 is selectively essential for CD4 T cell activation and effector function. Cell Metab 2014; 20: 61–72. 24930970 10.1016/j.cmet.2014.05.004 PMC4079750

[38] Rathmell JC , Farkash EA , Gao W *et al* IL‐7 enhances the survival and maintains the size of naive T cells. J Immunol 2001; 167: 6869–6876. 11739504 10.4049/jimmunol.167.12.6869

[39] Phan AT , Doedens AL , Palazon A *et al* Constitutive glycolytic metabolism supports CD8^+^ T cell effector memory differentiation during viral infection. Immunity 2016; 45: 1024–1037. 27836431 10.1016/j.immuni.2016.10.017 PMC5130099

[40] Gubser PM , Bantug GR , Razik L *et al* Rapid effector function of memory CD8^+^ T cells requires an immediate‐early glycolytic switch. Nat Immunol 2013; 14: 1064–1072. 23955661 10.1038/ni.2687

[41] van der Windt GJW , O’Sullivan D , Everts B *et al* CD8 memory T cells have a bioenergetic advantage that underlies their rapid recall ability. Proc Natl Acad Sci USA 2013; 110: 14336–14341. 23940348 10.1073/pnas.1221740110 PMC3761631

[42] O’Sullivan D , van der Windt GJW , Huang SC‐C *et al* Memory CD8^+^ T cells use cell‐intrinsic lipolysis to support the metabolic programming necessary for development. Immunity 2014; 41: 75–88. 25001241 10.1016/j.immuni.2014.06.005 PMC4120664

[43] van der Windt GJW , Everts B , Chang C‐H *et al* Mitochondrial respiratory capacity is a critical regulator of CD8^+^ T cell memory development. Immunity 2012; 36: 68–78. 22206904 10.1016/j.immuni.2011.12.007 PMC3269311

[44] Raud B , Roy DG , Divakaruni AS *et al* Etomoxir actions on regulatory and memory T cells are independent of Cpt1a‐mediated fatty acid oxidation. Cell Metab 2018; 28: 504–515. 30043753 10.1016/j.cmet.2018.06.002 PMC6747686

[45] O'Connor RS , Guo L , Ghassemi S *et al* The CPT1a inhibitor, etomoxir induces severe oxidative stress at commonly used concentrations. Sci Rep 2018; 8: 6289. 29674640 10.1038/s41598-018-24676-6 PMC5908836

[46] Xu A , Bhanumathy KK , Wu J *et al* IL‐15 signaling promotes adoptive effector T‐cell survival and memory formation in irradiation‐induced lymphopenia. Cell Biosci 2016; 6: 30. 27158441 10.1186/s13578-016-0098-2 PMC4858849

[47] Davenport B , Eberlein J , van der Heide V *et al* Aging of antiviral CD8^+^ memory T cells fosters increased survival, metabolic adaptations, and lymphoid tissue homing. J Immunol 2019; 202: 460–475. 30552164 10.4049/jimmunol.1801277 PMC6358025

[48] Menk AV , Scharping NE , Moreci RS *et al* Early TCR signaling induces rapid aerobic glycolysis enabling distinct acute T cell effector functions. Cell Rep 2018; 22: 1509–1521. 29425506 10.1016/j.celrep.2018.01.040 PMC5973810

[49] Chang C‐H , Curtis JD , Maggi LB *et al* Posttranscriptional control of T cell effector function by aerobic glycolysis. Cell 2013; 153: 1239–1251. 23746840 10.1016/j.cell.2013.05.016 PMC3804311

[50] Balmer ML , Ma EH , Bantug GR *et al* Memory CD8^+^ T cells require increased concentrations of acetate induced by stress for optimal function. Immunity 2016; 44: 1312–1324. 27212436 10.1016/j.immuni.2016.03.016

[51] Peng M , Yin N , Chhangawala S *et al* Aerobic glycolysis promotes T helper 1 cell differentiation through an epigenetic mechanism. Science 2016; 354: 481–484. 27708054 10.1126/science.aaf6284 PMC5539971

[52] Trebak M , Kinet J‐P . Calcium signalling in T cells. Nat Rev Immunol 2019; 19: 154–169. 30622345 10.1038/s41577-018-0110-7 PMC6788797

[53] Mak TW , Grusdat M , Duncan GS *et al* Glutathione primes T cell metabolism for inflammation. Immunity 2017; 46: 1089–1090. 28636957 10.1016/j.immuni.2017.06.009

[54] Wang R , Dillon CP , Shi LZ *et al* The transcription factor Myc controls metabolic reprogramming upon T lymphocyte activation. Immunity 2011; 35: 871–882. 22195744 10.1016/j.immuni.2011.09.021 PMC3248798

[55] Denton RM . Regulation of mitochondrial dehydrogenases by calcium ions. Biochim Biophys Acta 2009; 1787: 1309–1316. 19413950 10.1016/j.bbabio.2009.01.005

[56] Jacobs SR , Herman CE , MacIver NJ *et al* Glucose uptake is limiting in T cell activation and requires CD28‐mediated Akt‐dependent and independent pathways. J Immunol 2008; 180: 4476–4486. 18354169 10.4049/jimmunol.180.7.4476 PMC2593791

[57] Wieman HL , Wofford JA , Rathmell JC . Cytokine stimulation promotes glucose uptake via phosphatidylinositol‐3 kinase/Akt regulation of Glut1 activity and trafficking. Mol Biol Cell 2007; 18: 1437–1446. 17301289 10.1091/mbc.E06-07-0593 PMC1838986

[58] Saxton RA , Sabatini DM . mTOR signaling in growth, metabolism, and disease. Cell 2017; 169: 361–371. 10.1016/j.cell.2017.03.035 28388417

[59] Düvel K , Yecies JL , Menon S *et al* Activation of a metabolic gene regulatory network downstream of mTOR complex 1. Mol Cell 2010; 39: 171–183. 20670887 10.1016/j.molcel.2010.06.022 PMC2946786

[60] Man K , Miasari M , Shi W *et al* The transcription factor IRF4 is essential for TCR affinity–mediated metabolic programming and clonal expansion of T cells. Nat Immunol 2013; 14: 1155–1165. 24056747 10.1038/ni.2710

[61] Kidani Y , Elsaesser H , Hock MB *et al* Sterol regulatory element‐binding proteins are essential for the metabolic programming of effector T cells and adaptive immunity. Nat Immunol 2013; 14: 489–499. 23563690 10.1038/ni.2570 PMC3652626

[62] Marko AJ , Miller RA , Kelman A *et al* Induction of glucose metabolism in stimulated T lymphocytes is regulated by mitogen‐activated protein kinase signaling. PLoS ONE 2010; 5: e15425–e15427. 21085672 10.1371/journal.pone.0015425 PMC2978105

[63] Frauwirth KA , Riley JL , Harris MH *et al* The CD28 signaling pathway regulates glucose metabolism. Immunity 2002; 16: 769–777. 12121659 10.1016/s1074-7613(02)00323-0

[64] Klein Geltink RI , O’Sullivan D , Corrado M *et al* Mitochondrial priming by CD28. Cell 2017; 171: 385–397.e11. 28919076 10.1016/j.cell.2017.08.018 PMC5637396

[65] Cui G , Staron MM , Gray SM *et al* IL‐7‐induced glycerol transport and TAG synthesis promotes memory CD8^+^ T cell longevity. Cell 2015; 161: 750–761. 25957683 10.1016/j.cell.2015.03.021 PMC4704440

[66] Delgoffe GM , Kole TP , Zheng Y *et al* The mTOR kinase differentially regulates effector and regulatory T cell lineage commitment. Immunity 2009; 30: 832–844. 19538929 10.1016/j.immuni.2009.04.014 PMC2768135

[67] Sinclair LV , Howden AJM , Brenes A *et al* Antigen receptor control of methionine metabolism in T cells. eLife 2019; 8: e44210. 30916644 10.7554/eLife.44210 PMC6497464

[68] Sinclair LV , Rolf J , Emslie E *et al* Control of amino‐acid transport by antigen receptors coordinates the metabolic reprogramming essential for T cell differentiation. Nat Immunol 2013; 14: 500–508. 23525088 10.1038/ni.2556 PMC3672957

[69] Rodriguez PC , Quinceno DG , Ochoa AC *et al* L‐arginine availability regulates T‐lymphocyte cell‐cycle progression. Blood 2007; 109: 1568–1573. 17023580 10.1182/blood-2006-06-031856 PMC1794048

[70] Courtemanche C , Elson‐Schwab I , Mashiyama ST *et al* Folate deficiency inhibits the proliferation of primary human CD8^+^ T lymphocytes *in vitro* . J Immunol 2004; 73: 3186–3192. 10.4049/jimmunol.173.5.3186 15322179

[71] Bachem A , Makhlouf C , Binger KJ *et al* Microbiota‐derived short‐chain fatty acids promote the memory potential of antigen‐activated CD8^+^ T cells. Immunity 2019; 51: 285–297. 31272808 10.1016/j.immuni.2019.06.002

[72] Xu X , Araki K , Li S *et al* Autophagy is essential for effector CD8^+^ T cell survival and memory formation. Nat Immunol 2014; 15: 1152–1161. 25362489 10.1038/ni.3025 PMC4232981

[73] López‐Otín C , Blasco MA , Partridge L *et al* The hallmarks of aging. Cell 2013; 153: 1194–1217. 23746838 10.1016/j.cell.2013.05.039 PMC3836174

[74] Trifunovic A , Wredenberg A , Falkenberg M *et al* Premature ageing in mice expressing defective mitochondrial DNA polymerase. Nature 2004; 429: 417–423. 15164064 10.1038/nature02517

[75] Sahin E , Colla S , Liesa M *et al* Telomere dysfunction induces metabolic and mitochondrial compromise. Nature 2011; 470: 359–365. 21307849 10.1038/nature09787 PMC3741661

[76] de Magalhães J‐P , Curado J , Church GM . Meta‐analysis of age‐related gene expression profiles identifies common signatures of aging. Bioinformatics 2009; 25: 875–881. 19189975 10.1093/bioinformatics/btp073 PMC2732303

[77] Talens RP , Christensen K , Putter H *et al* Epigenetic variation during the adult lifespan: cross‐sectional and longitudinal data on monozygotic twin pairs. Aging Cell 2012; 11: 694–703. 22621408 10.1111/j.1474-9726.2012.00835.x PMC3399918

[78] Rubinsztein DC , Marino G , Kroemer G . Autophagy and aging. Cell 2011; 146: 682–695. 21884931 10.1016/j.cell.2011.07.030

[79] Tomaru U , Takahashi S , Ishizu A *et al* Decreased proteasomal activity causes age‐related phenotypes and promotes the development of metabolic abnormalities. Am J Pathol 2012; 180: 963–972. 22210478 10.1016/j.ajpath.2011.11.012

[80] Hekimi S , Lapointe J , Wen Y . Taking a “good” look at free radicals in the aging process. Trends Cell Biol 2011; 21: 569–576. 21824781 10.1016/j.tcb.2011.06.008 PMC4074523

[81] Gomes AP , Price NL , Ling AJY *et al* Declining NAD^+^ induces a pseudohypoxic state disrupting nuclear‐mitochondrial communication during aging. Cell 2013; 155: 1624–1638. 24360282 10.1016/j.cell.2013.11.037 PMC4076149

[82] Mather MW , Rottenberg H . The inhibition of calcium signaling in T lymphocytes from old mice results from enhanced activation of the mitochondrial permeability transition pore. Mech Age Dev 2002; 123: 707–724. 10.1016/s0047-6374(01)00416-x 11850032

[83] Johnson SC , Rabinovitch PS , Kaeberlein M . mTOR is a key modulator of ageing and age‐related disease. Nature 2013; 493: 338–345. 23325216 10.1038/nature11861 PMC3687363

[84] Franceschi C , Campisi J . Chronic inflammation (inflammaging) and its potential contribution to age‐associated diseases. J Gerontol Series A 2014; 69: S4–S9. 10.1093/gerona/glu057 24833586

[85] Palmer CS , Duette GA , Wagner MCE *et al* Metabolically active CD4^+^ T cells expressing Glut1 and OX40 preferentially harbor HIV during *in vitro* infection. FEBS Lett 2017; 591: 3319–3332. 28892135 10.1002/1873-3468.12843 PMC5658250

[86] Pawelec G . Immunosenenescence: role of cytomegalovirus. Exp Gerontol 2014; 54: 1–5. 24291068 10.1016/j.exger.2013.11.010

[87] Frasca D , Blomberg BB , Paganelli R . Aging, obesity, and inflammatory age‐related diseases. Front Immunol 2017; 8: 1745. 29270179 10.3389/fimmu.2017.01745 PMC5725402

[88] Thevaranjan N , Puchta A , Schulz C *et al* Age‐associated microbial dysbiosis promotes intestinal permeability, systemic inflammation, and macrophage dysfunction. Cell Host Microbe 2017; 21: 455–466. 28407483 10.1016/j.chom.2017.03.002 PMC5392495

[89] Acosta JC , Banito A , Wuestefeld T *et al* A complex secretory program orchestrated by the inflammasome controls paracrine senescence. Nat Cell Biol 2013; 15: 978–990. 23770676 10.1038/ncb2784 PMC3732483

[90] Rufer N , Brümmendorf TH , Kolvraa S *et al* Telomere fluorescence measurements in granulocytes and T lymphocyte subsets point to a high turnover of hematopoietic stem cells and memory T cells in early childhood. J Exp Med 1999; 190: 157–167. 10432279 10.1084/jem.190.2.157 PMC2195579

[91] Lanna A , Gomes DC , Muller‐Durovic B *et al* A sestrin‐dependent Erk‐Jnk‐p38 MAPK activation complex inhibits immunity during aging. Nat Immunol 2017; 18: 354–363. 28114291 10.1038/ni.3665 PMC5321575

[92] Phadwal K , Alegre‐Abarrategui J , Watson AS *et al* A novel method for autophagy detection in primary cells: impaired levels of macroautophagy in immunosenescent T cells. Autophagy 2012; 8: 677–689. 22302009 10.4161/auto.18935 PMC3405842

[93] Raz Y , Guerrero‐Ros I , Maier A *et al* Activation‐induced autophagy is preserved in CD4^+^ T‐cells in familial longevity. J Gerontol Series A 2017; 72: 1201–1206. 10.1093/gerona/glx020 PMC5861944 28486590

[94] Henson SM , Lanna A , Riddell NE *et al* p38 signaling inhibits mTORC1‐independent autophagy in senescent human CD8^+^ T cells. J Clin Invest 2014; 124: 4004–4016. 25083993 10.1172/JCI75051 PMC4151208

[95] Moskowitz DM , Zhang DW , Hu B *et al* Epigenomics of human CD8 T cell differentiation and aging. Sci Immunology 2017; 2. 10.1126/sciimmunol.aag0192 PMC5399889 28439570

[96] Ron‐Harel N , Notarangelo G , Ghergurovich JM *et al* Defective respiration and one‐carbon metabolism contribute to impaired naive T cell activation in aged mice. Proc Natl Acad Sci USA 2018; 115: 13347–13352. 30530686 10.1073/pnas.1804149115 PMC6310842

[97] Reed JR , Vukmanovic‐Stejic M , Fletcher JM *et al* Telomere erosion in memory T cells induced by telomerase inhibition at the site of antigenic challenge *in vivo* . J Exp Med 2004; 199: 1433–1443. 15148341 10.1084/jem.20040178 PMC2211820

[98] Utsuyama M , Wakikawa A , Tamura T *et al* Impairment of signal transduction in T cells from old mice. Mech Age Dev 1997; 93: 131–144. 10.1016/s0047-6374(96)01837-4 9089578

[99] Puleston DJ , Zhang H , Powell TJ *et al* Autophagy is a critical regulator of memory CD8+ T cell formation. eLife 2014; 3: e03706. 10.7554/eLife.03706 PMC4225493 25385531

[100] Lucas CL , Kuehn HS , Zhao F *et al* Dominant‐activating germline mutations in the gene encoding the PI(3)K catalytic subunit p110δ result in T cell senescence and human immunodeficiency. Nat Immunol 2013; 15: 88–97. 24165795 10.1038/ni.2771 PMC4209962

[101] Lee JH , Budanov AV , Karin M . Sestrins orchestrate cellular metabolism to attenuate aging. Cell Metab 2013; 18: 792–801. 24055102 10.1016/j.cmet.2013.08.018 PMC3858445

[102] Bengsch B , Johnson AL , Kurachi M *et al* Bioenergetic insufficiencies due to metabolic alterations regulated by the inhibitory receptor PD‐1 are an early driver of CD8^+^ T cell exhaustion. Immunity 2016; 45: 358–373. 27496729 10.1016/j.immuni.2016.07.008 PMC4988919

[103] Weng NP , Akbar AN , Goronzy J . CD28‐ T cells: their role in the age‐associated decline of immune function. Trends Immunol 2009; 30: 306–312. 19540809 10.1016/j.it.2009.03.013 PMC2801888

[104] Jeng MY , Hull PA , Fei M *et al* Metabolic reprogramming of human CD8^+^ memory T cells through loss of SIRT1. J Exp Med 2018; 215: 51–62. 29191913 10.1084/jem.20161066 PMC5748845

[105] Zhang H , Ryu D , Wu Y *et al* NAD^+^ repletion improves mitochondrial and stem cell function and enhances life span in mice. Science 2016; 352: 1436–1443. 27127236 10.1126/science.aaf2693

[106] Henson SM , Macaulay R , Riddell NE *et al* Blockade of PD‐1 or p38 MAP kinase signaling enhances senescent human CD8^+^ T‐cell proliferation by distinct pathways. Eur J Immunol 2015; 45: 1441–1451. 25707450 10.1002/eji.201445312

[107] Quinn KM , Fox A , Harland KL *et al* Age‐related decline in primary CD8^+^ T cell responses is associated with the development of senescence in virtual memory CD8^+^ T cells. Cell Rep 2018; 23: 3512–3524. 29924995 10.1016/j.celrep.2018.05.057

[108] Kim HJ , Nel AE . The role of phase II antioxidant enzymes in protecting memory T cells from spontaneous apoptosis in young and old mice. J Immunol 2005; 175: 2948–2959. 16116181 10.4049/jimmunol.175.5.2948

[109] Monti D , Salvioli S , Capri M *et al* Decreased susceptibility to oxidative stress‐induced apoptosis of peripheral blood mononuclear cells from healthy elderly and centenarians. Mech Ageing Dev 2000; 121: 239–250. 11164477 10.1016/s0047-6374(00)00220-7

[110] Schurich A , Pallett LJ , Jajbhay D *et al* Distinct metabolic requirements of exhausted and functional virus‐specific CD8 T cells in the same host. Cell Rep 2016; 16: 1243–1252. 27452473 10.1016/j.celrep.2016.06.078 PMC4977274

[111] Yanes RE , Zhang H , Shen Y *et al* Metabolic reprogramming in memory CD4 T cell responses of old adults. Clin Immunol 2019; 207: 58–67. 31279855 10.1016/j.clim.2019.07.003 PMC6827883

[112] Kishton RJ , Sukumar M , Restifo NP . Metabolic regulation of T cell longevity and function in tumor immunotherapy. Cell Metab 2017; 26: 94–109. 28683298 10.1016/j.cmet.2017.06.016 PMC5543711

[113] O'Sullivan D , Pearce EL . Targeting T cell metabolism for therapy. Trends Immunol. 2015; 36: 71–80. 25601541 10.1016/j.it.2014.12.004 PMC4323623

[114] Sukumar M , Liu J , Ji Y *et al* Inhibiting glycolytic metabolism enhances CD8^+^ T cell memory and antitumor function. J Clin Invest 2013; 123: 4479–4488. 24091329 10.1172/JCI69589 PMC3784544

[115] Barzilai N , Crandall JP , Kritchevsky SB *et al* Metformin as a tool to target aging. Cell Metab 2016; 23: 1060–1065. 27304507 10.1016/j.cmet.2016.05.011 PMC5943638

[116] Eikawa S , Nishida M , Mizukami S *et al* Immune‐mediated antitumor effect by type 2 diabetes drug, metformin. Proc Natl Acad Sci USA 2015; 112: 1809–1814. 25624476 10.1073/pnas.1417636112 PMC4330733

[117] Sun Y , Tian T , Gao J *et al* Metformin ameliorates the development of experimental autoimmune encephalomyelitis by regulating T helper 17 and regulatory T cells in mice. J Neuroimmunol 2016; 292: 58–67. 26943960 10.1016/j.jneuroim.2016.01.014

[118] Mu Q , Jiang M , Zhang Y *et al* Metformin inhibits proliferation and cytotoxicity and induces apoptosis via AMPK pathway in CD19‐chimeric antigen receptor‐modified T cells. Onco Targets Ther 2018; 11: 1767–1776. 29662316 10.2147/OTT.S154853 PMC5892609

[119] Chen C , Liu Y , Liu Y *et al* mTOR regulation and therapeutic rejuvenation of aging hematopoietic stem cells. Sci Signal 2009; 2: ra75. 19934433 10.1126/scisignal.2000559 PMC4020596

[120] Mannick JB , Del Giudice G , Lattanzi M *et al* mTOR inhibition improves immune function in the elderly. Sci Transl Med 2014; 6: 268ra179. 10.1126/scitranslmed.3009892 25540326

[121] Mannick JB , Morris M , Hockey H‐UP *et al* TORC1 inhibition enhances immune function and reduces infections in the elderly. Sci Trans Med 2018; 10. 10.1126/scitranslmed.aaq1564 29997249

[122] Araki K , Youngblood B , Ahmed R . The role of mTOR in memory CD8 T‐cell differentiation. Immunol Rev 2010; 235: 234–243. 20536567 10.1111/j.0105-2896.2010.00898.x PMC3760155

[123] Pearce EL , Walsh MC , Cejas PJ *et al* Enhancing CD8 T‐cell memory by modulating fatty acid metabolism. Nature 2009; 460: 103–107. 19494812 10.1038/nature08097 PMC2803086

[124] Mills KF , Yoshida S , Stein LR *et al* Long‐term administration of nicotinamide mononucleotide mitigates age‐associated physiological decline in mice. Cell Metab 2016; 24: 795–806. 28068222 10.1016/j.cmet.2016.09.013 PMC5668137

[125] Goldberg EL , Romero‐Aleshire MJ , Renkema KR *et al* Lifespan‐extending caloric restriction or mTOR inhibition impair adaptive immunity of old mice by distinct mechanisms. Aging Cell 2014; 14: 130–138. 25424641 10.1111/acel.12280 PMC4326902

